# Antioxidant components of naturally-occurring oils exhibit marked anti-inflammatory activity in epithelial cells of the human upper respiratory system

**DOI:** 10.1186/1465-9921-12-92

**Published:** 2011-07-13

**Authors:** Meixia Gao, Anju Singh, Kristin Macri, Curt Reynolds, Vandana Singhal, Shyam Biswal, Ernst W Spannhake

**Affiliations:** 1 Health Effects Assessment Laboratory, Department of Environmental Health Sciences, The Johns Hopkins University Bloomberg School of Public Health, Baltimore, Maryland 21205, USA

## Abstract

**Background:**

The upper respiratory tract functions to protect lower respiratory structures from chemical and biological agents in inspired air. Cellular oxidative stress leading to acute and chronic inflammation contributes to the resultant pathology in many of these exposures and is typical of allergic disease, chronic sinusitis, pollutant exposure, and bacterial and viral infections. Little is known about the effective means by which topical treatment of the nose can strengthen its antioxidant and anti-inflammatory defenses. The present study was undertaken to determine if naturally-occurring plant oils with reported antioxidant activity can provide mechanisms through which upper respiratory protection might occur.

**Methods:**

Controlled exposure of the upper respiratory system to ozone and nasal biopsy were carried out in healthy human subjects to assess mitigation of the ozone-induced inflammatory response and to assess gene expression in the nasal mucosa induced by a mixture of five naturally-occurring antioxidant oils - aloe, coconut, orange, peppermint and vitamin E. Cells of the BEAS-2B and NCI-H23 epithelial cell lines were used to investigate the source and potential intracellular mechanisms of action responsible for oil-induced anti-inflammatory activity.

**Results:**

Aerosolized pretreatment with the mixed oil preparation significantly attenuated ozone-induced nasal inflammation. Although most oil components may reduce oxidant stress by undergoing reduction, orange oil was demonstrated to have the ability to induce long-lasting gene expression of several antioxidant enzymes linked to Nrf2, including HO-1, NQO1, GCLm and GCLc, and to mitigate the pro-inflammatory signaling of endotoxin in cell culture systems. Nrf2 activation was demonstrated. Treatment with the aerosolized oil preparation increased baseline levels of nasal mucosal *HO-1 *expression in 9 of 12 subjects.

**Conclusions:**

These data indicate that selected oil-based antioxidant preparations can effectively reduce inflammation associated with oxidant stress-related challenge to the nasal mucosa. The potential for some oils to activate intracellular antioxidant pathways may provide a powerful mechanism through which effective and persistent cytoprotection against airborne environmental exposures can be provided in the upper respiratory mucosa.

## Background

Inflammation in the respiratory system related to tissue oxidant stress is common to a wide variety of airborne exposures and infections. Among well-described environmental exposures are the oxidant pollutants, ozone and nitrogen dioxide, ambient particulate matter, and cigarette smoke [1-6]. Many acute and chronic inflammatory diseases of the airways are also associated with oxidant stress and include chronic obstructive pulmonary disease (COPD), asthma, chronic sinusitis, viral and bacterial infections, and idiopathic pulmonary fibrosis [7-14].

Studies support the concept that the upper respiratory system plays an important protective role in many of these types of challenges. In the case of chemical agents, this is achieved by the capture and neutralization of foreign agents in the inspired airstream, limiting their impact on lower airway structures [15]. It has also been demonstrated that the nose can serve as a repository for inhaled viral and bacterial pathogens where they can be eliminated or held in check by immune defenses, thereby reducing the risk and/or severity of lower airway infections [16-19].

Evidence indicates that inherent antioxidant and other protective defenses in the tissues of the upper and lower respiratory structures mitigate pulmonary inflammation and that enhancement of these protective pathways can reduce tissue damage, immune responses and morbidity [20,21]. However, little is known about mechanisms through which nasal antioxidant processes might be augmented and, if so, to what extent such augmentation would be effective as an intervention. As the primary cell of interface between the internal and external environments, the mucosal epithelial cell has long been the focus of much attention as a mediator of external stimuli and facilitator of both innate and acquired immune defenses in the respiratory tract [22,23]. Respiratory epithelial cells are known to initiate the release of a cascade of proinflammatory mediators through redox signaling [8,24,25]. In addition, these cells have the capacity to exhibit up-regulation of very effective antioxidant defense mechanisms involving the secretion of decoy oxidant targets, as well as the synthesis of a broad spectrum of antioxidant enzymes [26,27]. Agents with the ability to enhance antioxidant pathways and interfere with proinflammatory signaling in the upper respiratory epithelial mucosa could enhance the protection afforded by these air passages.

The current studies were undertaken to determine if natural oils with reported antioxidant activities might represent a well-tolerated and potentially effective means through which to enhance innate protective mechanisms in the nose. For the purposes of this investigation, focus was directed on a formulation containing five of these oils - coconut, orange, aloe, peppermint and vitamin E - for which the literature provides evidence of their direct action as reducing agents, but does not address other potential pathways of their antioxidant activity. In the case of coconut oil, its phenolic acid constituents have been proposed as the primary sources of its oxidant species scavenging activity [28]. Various fractions of orange oil have been shown to contain flavonoids and phenolic acids, as well as constituent aldehydes, such as citronellal, decanal, and terpine alcohol constituents, such as linalool. These components have been demonstrated to exert antioxidant activity through direct scavenging of hydroxyl and other radicals [29,30]. A large number of phenolic constituents are also found in oil derived from Aloe and have been shown to be primarily responsible for its superoxide and hydroxyl radical and hydrogen donating capacity [31,32]. The phenolic constituents of oil derived from peppermint leaves include fatty acids, and flavonoids that are very efficient scavengers of oxidant radicals, especially hydroxyl radical [33,34]. The well-described antioxidant activity of vitamin E (tocopherol) is primarily due to the capacity of its heterocyclic chromanol ring to donate phenolic hydrogen to peroxyl radicals, a key process in protecting the integrity of lipid membranes [35]. Soy oil was used as a carrier oil because of its reported high oxidative stability [36]. For these studies, the actions of a mixture of these oils administered by aerosol spray were investigated in human subjects and by direct application in human epithelial cell culture systems. The goals were (1) to investigate if preventive treatment with the oil mixture could be demonstrated to abrogate *in vivo *pathophysiologic responsiveness to a controlled oxidant challenge in the nose and (2) to utilize human epithelial cell culture to identify the presence of unique antioxidant activity beyond the scavenging of reactive oxidant species and investigate the mechanism through which such protective effect might be mediated within the cells of the airway epithelium.

## Methods

### Preparation of Test Compounds

The oil-based preparation used in the present study was supplied by Global Life Technologies Corp (GLT) (Chevy Chase, MD) and is a member of their Nozin^® ^brand product line. The study formulation contains the following components: soy oil - 69.18%; coconut oil - 20.00%; orange oil - 4.90%; aloe vera oil - 4.90%; peppermint oil - 0.75%; vitamin E - 0.27%. All components of the test formulation are USP-grade and have been individually evaluated and identified by the FDA to fall under the Generally Recognized as Safe classification. This formulation has been demonstrated to be without irritating or inflammatory effects in an *in vivo *mammalian mucosal test system in studies carried out on behalf of GLT by North American Science Associates, Inc., an independent FDA-approved safety testing agency. The oil-based preparation was administered as supplied in both *in vivo *and *in vitro *experiments, as described below.

For the human nasal studies, sterile water without additives, containing 0.75% peppermint oil as a scented masking agent, was selected for use as the sham test agent. Because saline, itself, has been reported to reduce inflammatory cell number in the nose [37], water was considered to represent an appropriate vehicle against which to compare the oil-based preparation.

### Subjects

This study was conducted as prescribed by the research protocol reviewed and approved by the Institutional Review Board of the Johns Hopkins Bloomberg School of Public Health. The study employed a single blind cross-over design, as described in the treatment and exposure protocol, below. Nine healthy adult men and women (22 to 40 years of age) were recruited into the ozone exposure study after obtaining informed consent (Table 1). Subjects were excluded if they had a history of chronic respiratory disease, cardiovascular disease or upper respiratory infection during the previous four weeks, if they were "smokers" or if they indicated an inability to sustain light exercise for at least 30 min. "Non-smokers" were defined as those individuals with a lifetime total of fewer than 3 pack-years plus abstinence from smoking of at least one year prior to the study. Subjects were required to refrain from taking prescription and non-prescription anti-inflammatory medications for the week prior to, and for the duration of, the 3-week study period. One subject was removed from the study after the initial nasal lavage indicated the presence of very high numbers of leukocytes in the nose (>100,000/ml), suggesting the presence of a latent upper respiratory infection. A second subject withdrew himself for reasons unrelated to the study.

**Table 1:** Exposure of Healthy Subjects to Ozone

				Change in Inflammatory Cell Counts* Pre- to Post-Ozone	
				
Subject #	Age (Yrs)	Gender		Arm 1 (Sham Treatment)	Arm 2 (Test Oil Treatment)
1	24	M		1099	-110
2	33	M		2503	-314
4	25	M		1825	34
5	30	F		666	-1209
6	27	M		70	-2581
7	22	M		701	-435
9	28	M		516	-361
				
			Mean (SEM)	1054 (317)	-711 (346)†

*Inflammatory cell counts expressed as number/ml lavage fluid returned.

†Significantly different from sham treatment (P < 0.001) by paired t analysis.

Consistent with Institutional Review Board approval and following the same exclusion and informed consenting procedures described above, a second cohort of 12 different healthy adult subjects (9 men and 3 women) 22 to 62 years of age were recruited to assess the effects of the oil preparation on baseline antioxidant gene expression in the nasal epithelium (Table 2).

**Table 2:** Activation of Nrf2 by Components of the Natural Oil Preparation

Component*	Relative Luciferase Activity**	
	
	Mean	SEM
Medium Control	7.0	0.6
Soy Oil Vehicle	7.5	0.5
Sulforaphane (100 um)	41.8†	1.3
Mixed Oil Preparation	40.8†	5.2
Coconut Oil (20%)	6.6	0.8
Orange Oil (5%)	52.5†	9.2
Aloe Oil (5%)	5.7	0.7
Peppermint Oil (0.75%)	5.7	0.9
Vitamin E (0.25%)	5.0	0.7

Mean values from 4 separate experiments in which duplicate cultures were treated.

*Individual oil percentages represent v/v in soy oil: treatment duration = 15 min, except sulforaphane = 12 hr.

**Activation of Nrf2 expressed as relative luciferase luminescence per mg cellular protein.

†Significantly increased above Medium Control value, P < 0.001, Mann-Whitney Rank Sum Test.

### Cells

#### BEAS-2B Cells

Cells of the BEAS-2B human bronchial epithelial cell line were obtained from the American Type Culture Collection (ATCC, Bethesda, MD). Cultures were expanded by growth on T-75 plastic flasks in DMEM/F-12 (1:1) medium (Invitrogen, Grand Island, NY) and seeded on 6- or 12-well Falcon filter inserts ( 0.4 μm pore size; Becton Dickinson, Franklin Lakes, NJ) and grown to confluence with the same medium above and below prior to treatment.

#### H23 Cells

Cells of the NIH-H23 human lung cell line were purchased from the American Type Culture Collection. H23 cells were transfected with a plasmid vector (pGL3 vector with a minimal promoter) purchased from Promega Corporation, Madison, WI, expressing the firefly luciferase gene driven by a minimal TATA-like promoter. Upstream to the promoter, a short DNA fragment containing the Nrf2 binding site found in the NQO1 gene promoter was cloned, as previously described in BEAS-2B cells [38]. H23 cells expressing the reporter plasmid were selected using blasticidin as the antibiotic. Several clones were screened using the luciferase assay and one clone exhibiting maximum luciferase activity was selected for detailed characterization of its Nrf2 activation profile. Sulforaphane, a naturally-occurring isothiocyanate known to activate Nrf2 [39] was used to demonstrate that Nrf2 dependent luciferase reporter activity in the H23-ARE-luciferase cells was dose-dependent and linked to downstream antioxidant enzyme gene activation [38]. These cells were cultured and seeded on filter inserts, as described above, and used in all assessments of Nrf2 activation in the present study.

### Treatment and Exposure of Subjects to Ozone

**Figure 1:**
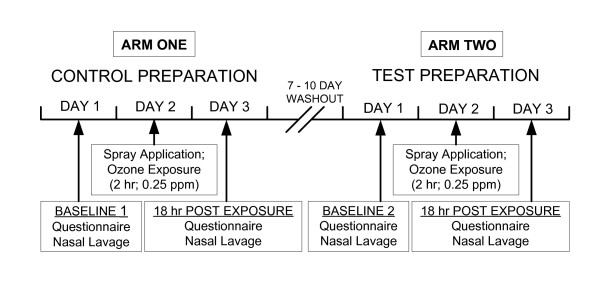
**Depiction of the ozone exposure intervention protocol**.

**Figure 2:**
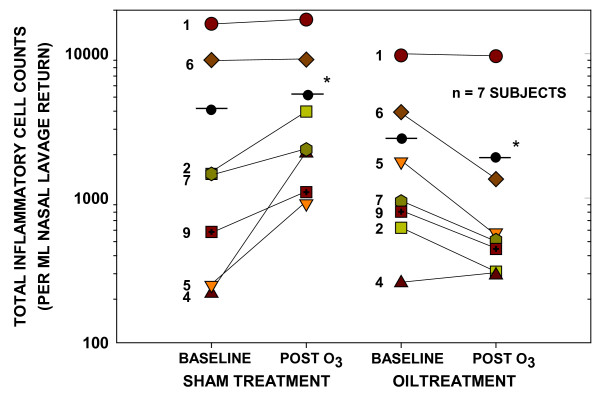
**Intervention in oxidant pollutant exposure-induced inflammation at 18 hrs by topical application of the oil preparation**. Individual data showing the upper respiratory inflammatory responses of subjects exposed to ozone (0.25 ppm, 2 hr) when pretreated with 50 μl of scented sterile water (sham) or a mixture of natural oils administered by aerosol spray to each nostril. Each subject is represented by the same symbol in both arms of the study; numbers correspond to subject numbers in Table 1. Points connected by dashed lines represent means of each group. * indicates significant difference from baseline (P < 0.05) by paired t analysis.

The human subjects component of the study was carried out in the Health Effects Assessment Laboratory (HEAL) in the Department of Environmental Health Sciences of the Bloomberg School of Public Health. The purpose of this study was to test the hypothesis that oil treatment would mitigate ozone-induced upper respiratory system neutrophil inflammation. A single blind, non-randomized design was chosen to enable the identification and elimination from further unnecessary participation any individuals who were unresponsive to this level of ozone exposure. The masking scent in both sham and test preparations kept subjects blinded to the treatments in each arm. The study design is depicted in Figure 1. On the first day of Arm 1 of the protocol, the absence of baseline inflammation was confirmed in each subject by determining that inflammatory cell concentrations fell within normal limits (<20,000 cells/ml nasal lavage) (Figure 2). On the second day of Arm 1, the aqueous control preparation containing 0.75% peppermint oil as a masking agent was administered in a single-blinded manner as a single 50 μl application in each nostril using a metered spray applicator (model VP7/50 18/415 + poussoir 232 NA/B) manufactured by Aptar (Le Vaudreuil, France). Immediately following nasal treatment, subjects were exposed to 0.25 ppm O_3 _for 120 min. with alternating 30 min periods of rest and light exercise consisting of slowly walking on a treadmill. Exposures took place in a temperature- and humidity-controlled chamber as previously described [40]. To optimize upper respiratory targeting, subjects were visually monitored after being instructed to chew gum with a closed mouth for the duration of the exposure period. Eighteen hours following exposure, subjects underwent nasal lavage to assess post-exposure. After a 7-10 day washout period, a second 3-day study period was repeated in Arm 2 following the same procedures, but associated with the nasal spray application of the oil-based test agent.

### Assessment of Nasal Inflammation

Nasal lavage was carried out according to a standardized procedure. With the subject seated in a chair and the head tilted backwards, 5 ml of 37°C Ringer's lactate was instilled by pipette into each nostril. After 5-10 seconds, the head was brought forward and the fluid expelled into a basin by gentle blowing. This procedure was repeated 4 times. Following centrifugation, the cells from all 4 tubes were pooled by re-suspension in phosphate buffered saline for cellular analysis.

Counts of inflammatory cells were made using a hemocytometer and calculated as total inflammatory cells per ml of nasal lavage return. Return volumes, which averaged 84% of the 40 ml instilled volume, were very consistent within each subject and were used to normalize the inflammatory cell return. In healthy adults without respiratory disease or allergic symptoms, ozone exposure elicits a predominantly polymorphonuclear neutrophilic (PMN) inflammatory response [41]. In the present study, PMNs comprised greater than 95% of the inflammatory cells recovered in nasal lavage fluid. Thus, the total number of leukocytes retrieved by lavage was used as an index of nasal inflammation.

Cell-free supernatant from nasal lavage samples was stored at -80°C prior to assay for the presence of the inflammatory mediators IL-6 and IL-8 by ELISA (R & D Systems, Minneapolis, MN).

Nasal symptoms prior to and at eighteen hours post exposure in each of the two arms were scored following a standard procedure [42,43] by having the subjects make a mark on a horizontal 100 mm line indicating the level of the symptom described, with the least sensation at the far left and the most at the far right. Scores were determined by measuring the distance in mm from the left end of the line and the change in numerical values between the two arms were compared.

### Assessment of Nasal Epithelial Gene Expression

Collection of nasal mucosal epithelial cells was made from the upper and lower aspects of the inferior medial turbinates of the right and left nostrils using a nasal mucosal curette (Rhino-probe^®^). Epithelial biopsy samples were taken prior to and 8 hours following administration of the oil-based test agent or the scented control preparation utilized in the ozone study. Using a metered spray applicator, 50 microliters of each of the two agents was administered in a single-blinded and random manner to one or the other of the two nostrils. Using this design, each turbinate provided its own baseline value for gene expression and the two agents were tested simultaneously in the same individual. Preliminary experiments in several subjects demonstrated that prior sampling on the turbinate at a site distant from the second sample site had no effect on baseline expression of the heme oxygenase-1 (*HO-1*) target gene in the second sample in the absence of treatment (first to second expression ratio = 0.96 ± 0.06; mean ± SEM, *n *= 8 sample pairs from 9 subjects). Biopsy samples were frozen in liquid nitrogen and stored for RNA extraction and PCR analysis as described below.

### Treatment of cells in culture

After ensuring that the surfaces of BEAS-2B epithelial cell cultures were free of liquid, 200 μl of control agent (HBSS or soy oil, as indicated) or test oil preparation were added to the apical surfaces and evenly distributed by rotation. It was found that the soy oil component of the test preparation was indistinguishable from HBSS as a negative control and, thus, soy oil was used in the majority of cell culture studies as the control; ratio of threshold cycle for *HO-1 *gene expression HBSS:soy = 1:1.01 ± 0.09 (±SD; *n *= 6). After treatment, the cultures were returned to the incubator for 15 min. prior to removal of the treatment fluids by suction. The surfaces were then gently washed twice with 500 μl of warmed (37°C) HBSS, and the cultures were returned to the incubator for the designated periods of time prior to extraction of RNA or protein. In two series of experiments, control and oil-treated cells underwent further challenge at 12 hours with lipopolysaccharide (LPS, 3 μg/ml medium, *Escherichia coli*, serotype 055.B5 - Sigma.) for 4 hours prior to RNA extraction. The removal of treatment oil by suction and the repeated aqueous wash and removal of wash fluid and floating oil by suction ensured the complete removal of oil from the cultures prior to subsequent treatment. In the four sets of experiments in which activation of Nrf2 by individual treatment oil constituents and four sets in which the dose-dependency of Nrf2 activation by orange oil was assessed, duplicate cultures were extracted for measurement of luciferase activity at the times indicated, as described below.

### Determination of Gene and Protein Expression

#### Real Time RT-PCR

Total RNA was extracted from cultured cells and from nasal mucosal epithelial cells obtained by biopsy using the RNeasy kit (Qiagen) and was quantified by UV absorbance spectrophotometry. The reverse transcription reaction was performed by using the high capacity cDNA synthesis kit (Applied Biosytems) in a final volume of 20 μl containing 1 μg of total RNA, 100 ng of random hexamers, 1X reverse transcription buffer, 2.5 mM MgCl_2_, 1 mM dNTP, 20 units of multiscribe reverse transcriptase, and nuclease free water. Quantitative real time RT-PCR analyses of Human heme oxygenase-1 (*HO-1)*, NAD(P)H:quinone oxidoreductase 1 (*NQO1)*, glutamate cysteine ligase-modulatory subunit *(GCLm)*, glutamate cysteine ligase-catalytic subunit *(GCLc)*, and tumor necrosis factor alpha (TNFα) were performed on cell and nasal biopsy extracts using primers and probe sets from Applied Biosystems. Assays were performed by using the ABI 7000 Taqman system (Applied Biosystems). β-*actin *was used for normalization.

#### Western Blot Analysis

To obtain total protein lysates, cells were lysed in RIPA buffer containing Halt Protease Inhibitor cocktail (Pierce, Rockford, Illinois, United States) and centrifuged at 12,000 *g *for 15 min at 4°C. Protein concentrations of the supernatant were measured using Bio-Rad protein assay (Bio-Rad, CA). To detect the translocation of Nrf2 protein to the nucleus, nuclear protein was isolated using the NE-PER protein isolation kit (Pierce, Rockford, IL). For immunoblot analysis, 20 μg of total protein lysate or 20 μg of nuclear protein lysate was resolved on 12% SDS-PAGE gels. Proteins were transferred onto PVDF membranes and blocked with PBS- Tween (0.1% Tween-20 in PBS, pH 7.2) supplemented with 5% low fat milk powder (w/v) for 2 hr at room temperature. All primary antibodies were diluted in PBS-Tween (0.1%) with 5% nonfat dry milk and incubated overnight at 4°C. Following antibodies were used for immunoblotting: anti-HO1 (Abcam), anti-NQO1 (Novus Biologicals), anti-GCLm, and anti-GAPDH (Imgenex, Sorrento Valley, CA), anti-Nrf2 and anti-lamin B (Santa Cruze Biotechnology (Santa Cruze, CA). After washing the primary antibody, the membranes were incubated with horseradish peroxidase conjugated anti-rabbit, anti-mouse or anti-goat antibody (~1:2500 in 0.1% Tween-20, with 5% low fat milk powder (w/v) for 1 hr at room temperature. Membranes were again washed with PBS-Tween (0.1%) and secondary antibodies were visualized by enhanced chemiluminescence detection system (Amersham Biosciences, NJ). Densitometric measurement of individual target protein lots were normalized to GAPDH or lamin B and quantified using the Image J (NIH) software package for graphic display.

### Determination of Nrf2 Activation

Changes in activation of the Nrf2 transcription factor in cultured H23 ARE cells in response to treatment were determined as previously described [44]. In brief, cells grown to 70% confluence on 12-well inserts were treated as indicated for 15 min and incubated for 12 hr at 37°C. Rinsed cells were fully lysed and luciferase luminescence was generated using the E4030 Luciferase Assay System (Promega) and detected with a TD-20/20 Luminometer (Turner Designs). Luminescence was normalized to the protein content of each lysate as determined using the Bio-Rad Protein Assay System and Microplate Reader. Nrf2 activation levels were expressed as Relative Luminescence Units/ug protein.

### Statistics

Nasal lavage and biopsy data were tested for differences between control and oil-treatments using paired-t analyses. Comparisons of cell culture data were made using Student's t analyses. In instances of a lack of normality, the Wilcoxon Signed Rank Test was used. In all cases, P values < 0.05 were considered significant. Statistical analyses were carried out with SigmaStat Statistical software (Jandel Scientific, San Rafael, CA).

## Results

### Ozone-induced nasal inflammation

In the majority of individuals, the typical response of exposure of the upper and lower respiratory epithelium to ozone is inflammation and an influx of inflammatory cells, especially PMNs, to mucosal and luminal regions. This process is mediated by the oxidant stress-related release of pro-inflammatory mediators, primarily IL-8, by epithelial cells. As a means to determine if administration of the oil preparation could afford protection against this example of oxidant-induced inflammation in the upper respiratory system, the effect of pretreatment with the oil was compared to that of sham control. As assessed by nasal lavage, controlled exposure to 0.25 ppm ozone for 2 hr resulted in nominal to 9-fold increases in inflammatory cell influx in seven subjects undergoing sham pretreatment (Figure 2). Differential cell counts showed these cells to be > 96% PMNs with occasional mononuclear and infrequent eosinophilic cells. This increase in inflammatory response was statistically significant within this treatment group. In the same subjects undergoing pretreatment with the aerosolized oil preparation, the ozone-induced increase in inflammatory cells in the nasal lavage was completely inhibited. Moreover, post-exposure cell numbers were statistically reduced below those present at baseline prior to the exposure (Figure 2). This observation suggested that a mechanism beyond simple blockade of ozone access to the tissues or scavenging of ozone-derived reactive species, perhaps involving direct reduction of inflammatory signaling, was initiated in tissues undergoing oil treatment. Comparison of the two treatment regimens based on pre- to post-ozone changes in inflammatory cell counts demonstrated that administration of the oil preparation significantly reduced the pro-inflammatory response of the subjects to ozone exposure (Table 1). Average lavage return was not different in the two treatment arms (sham: 33.4 ml; oil: 33.7 ml).

ELISA determinations of Interleukins 6 and 8 indicated that these inflammatory mediators were not detectable in nasal lavage samples at 18 hr post ozone exposure. The time of the nasal lavage was selected to coincide with expected peak neutrophil presence in the nasal airspace at 18 hours following the short, 2 hr exposure period. It is likely that this time point was too late to detect the presence of these early mediators in the nasal lavage.

Consistent with reduced levels of tissue inflammation as assessed by cellular influx at 18 hr post-exposure, symptom scores for "ease of airflow through the nose" at that time were significantly greater in ozone-exposed subjects following pretreatment with the oil preparation when compared to sham treatment (P < 0.05). This was the only nasal symptom measure to show significant change during the study.

### Inhibition of endotoxin-induced pro-inflammatory gene expression by the oil preparation

In order to pursue the possibility that the reduction in nasal inflammatory response to ozone exposure was not due simply to a barrier effect of the oil preparation on the nasal epithelial tissues, the anti-inflammatory effect of oil treatment was tested in cultures of BEAS-2B cells utilizing the bacterial endotoxin lipopolysaccharide (LPS) rather than ozone to induce pro-inflammatory signaling. Twelve hours following a 15 minute pretreatment of cell cultures with HBSS (control) or the oil preparation, cells were exposed to 3 μg/ml of LPS for 4 hours. At the end of the exposure period, real-time PCR was used to assess gene expression of *TNFα*, an early proinflammatory cytokine that is elevated during LPS-induced inflammation. *TNFα *transcript levels were normalized to those of actin. Two separate experiments were performed, utilizing triplicate cultures per treatment group; fold change data are presented as mean ± SD. There was no difference in relative expression of *TNFα *between control cells and those treated with the oil-based preparation alone (control: 1.1 ± 0.54; oil: 0.51 ± 0.19). In sham-pretreated cultures, LPS increased expression of *TNFα *by 81-fold, compared to that in unchallenged controls (80.6 ± 23.4). In contrast, in oil pre-treated cells, the fold-change in proinflammatory signaling induced by LPS exposure was reduced by more than 50% (33.4 ± 3.6 fold compared to controls). These data provided preliminary support of the notion that one or more components of the oil preparation may act by directly inducing antioxidant/anti-inflammatory pathways within the respiratory epithelium.

### Kinetics of antioxidant gene expression induced by the oil preparation

**Figure 3:**
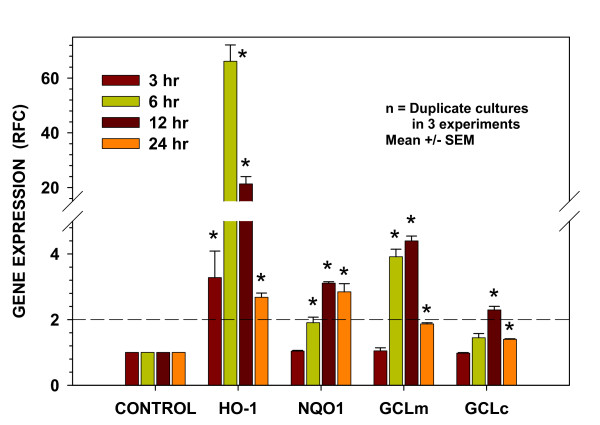
**Treatment of cells with the mixed oil preparation increases expression of oxidant-protective pathways with differing activation kinetics**. Time-courses of expression of antioxidant genes *HO-1*, *NQO1*, *GCLm*, and *GCLc *in cells of the BEAS-2B human bronchial epithelial line at designated times following the 15 min treatment period. Data are presented as fold change from time-matched soy oil controls after normalization to the expression of actin. Presented are results from three separate experiments. The dashed line indicates the 2-fold level of increased expression as a reference for potential biological significance. Results are expressed as mean ± SEM. * indicates significantly different from time-matched soy oil controls (P < 0.05) by Student's t test.

To investigate the effects of the oil preparation on the global system of antioxidant genes induced within the respiratory epithelium by the Nrf2 transcription factor, representative gene targets of Nrf2 were selected for study. The kinetics of gene expression induced by 15 min of oil treatment were investigated in cells of the BEAS-2B line following extraction at 3, 6, 12, and 24 hours after treatment. As seen in Figure 3, *HO-1 *exhibited a 3.5-fold increase in expression at 3 hours that reached more than 60-fold by 6 hours. After remaining at a 20-fold level of elevation for up to 12 hours, *HO-1 *expression returned toward its time-matched control, but remained elevated by more than 2-fold at 24 hours. In contrast, neither *NQO1 *nor *GCLc *expression increased by more than 2.0-fold until 12 hours post treatment, when they increased 3.1- and 2.2-fold, respectively. The increase in *NQO1 *expression above 2-fold remained through 24 hrs. Expression of the modulatory sub-unit of *GCL *was likewise delayed relative to *HO-1*, but remained at or above a 4-fold level of activation at 6 and 12 hours. These data provide evidence that one or more constituents within the oil preparation activate Nrf2.

### Activation of Nrf2 by components of the oil preparation

In order to identify the source of Nrf2 activation within the oil preparation, constituent oils were individually prepared in soy oil carrier in the same concentrations as in the combined preparation. Medium and soy oil were used as negative controls and the known Nrf2 activator, sulforaphane, and the mixed oil preparation were used as positive controls. With the exception of sulforaphane, cultures of H23 reporter cells were treated for 15 min with the test preparations, incubated, and assayed for luciferase luminescence at 12 hr post-treatment, as described in Methods. In all cell culture studies, the water soluble Nrf2 activator, sulforaphane, dissolved in cell culture medium was allowed to remain on the cultures for the entire 12 hr incubation period prior to assay. Preliminary experiments had shown that treatment of the cells for the 15 min exposure period was inadequate to cause significant activation of Nrf2 above that seen in medium or soy-treated controls. In contrast, 12 hr treatment resulted in 5-fold higher activation levels (15 min: 8.4 ± 1.5 RLA vs. 12 hr: 41.8 ± 1.7 RLA, Mean + SD). The results of the screening of mixed oil preparation components, presented in Table 2, indicate that orange oil was the apparent single source of Nrf2 activation within the preparation of antioxidant natural oils, inducing levels of activation comparable to those of the original mixed oil preparation.

### Nrf2 Activation by orange oil

**Figure 4:**
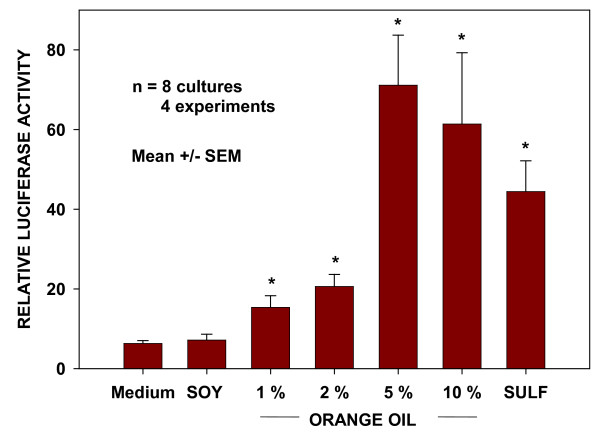
**Activation of Nrf2 by orange oil**. Dose-related activation of Nrf2 based on luciferase luminescence in cells containing the ARE-luciferase reporter construct. Activity was assessed at 12 hr following the 15 min treatment period. * indicates significantly different from soy oil-treated control cultures (P < 0.05) by Student's t test.

The dose-related activation of Nrf2 by orange oil was assessed in cultures of BEAS-2B cells. Cell cultures were treated for 15 min with a range of concentrations of orange oil in soy oil from 1% to 10% and assayed at 6 hr post treatment. Culture medium, soy oil and sulforaphane were used as controls. The data depicted in Figure 4 demonstrate a dose-response relationship with reaching a maximum at 5%, with no further increase at 10%.

### Assessment of cellular toxicity

**Figure 5:**
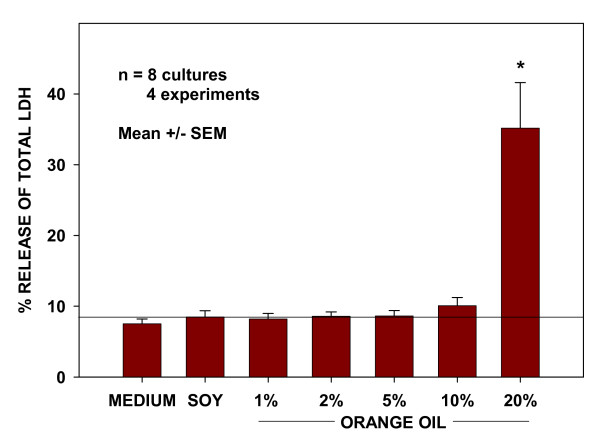
**Absence of LDH release in response to effective concentrations of orange oil**. LDH release from BEAS-2B cells used as a measure of increased membrane permeability in response to control and oil treatments. * indicates significantly different from soy oil control cultures (P < 0.05) by Student's t test.

Because cellular toxicity-related oxidant stress associated with chemical exposures can initiate Nrf2 activation, a series of experiments were undertaken to assess the potential toxic effects of the orange oil. The release of LDH by cells was used as a sensitive marker of toxicity. BEAS-2B cells were treated as before with controls and a range of orange oil concentrations from 1% to 20% and assayed for total LDH release during the subsequent 6 hr period. The data presented in Figure 5 indicate no measurable differences in LDH release in the range from 1% to 10% compared to control treatments, suggesting that the observed activation of Nrf2 in that dose range was associated with activation mechanisms unrelated to cellular toxicity.

### Translocation of Nrf2 to the nucleus

**Figure 6:**
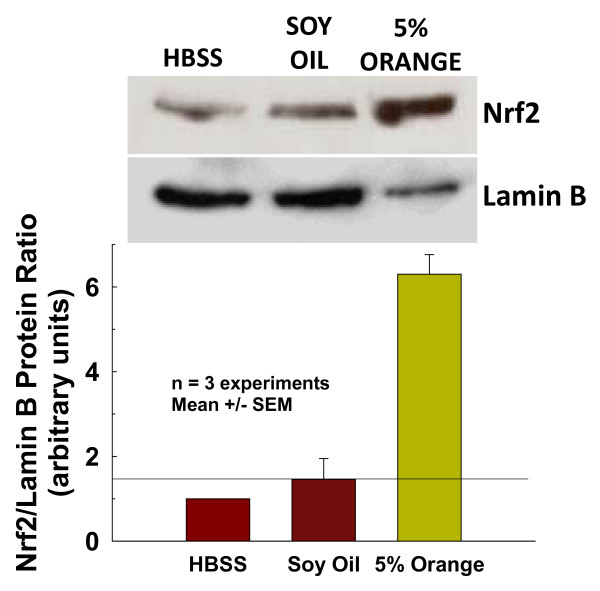
**Rapid translocation of Nrf2 to the nucleus**. Increase in levels of Nrf2 in the nuclear protein fraction of cells observed at 2 hr following treatment with orange oil compared to buffer and soy oil-treated controls. Representative blots from one of three separate experiments are shown above. Mean Nrf2 blot densities normalized to those of their corresponding nuclear lamin B for all three experiments presented below. Bars represent mean ± SEM.

To provide further evidence that the effects of orange oil treatment were Nrf2-associated, Western blot analysis was used to determine translocation of Nrf2 protein to the nucleus of BEAS-2B cells in 3 separate experiments, all of which showed similar results. Figure 6 shows representative blots from of one of these experiments and mean data from all three, which demonstrated a greater than 4-fold increase in the presence of Nrf2 protein in the nuclei of cells at 2 hr following treatment with 5% orange oil in soy compared to soy oil-treated controls. The nuclear protein lamin B was used to normalize protein loading. These data are consistent with the rapid translocation of Nrf2 from the cytoplasm to the nucleus following orange oil treatment.

### Kinetics of orange oil-induced antioxidant gene expression

**Figure 7:**
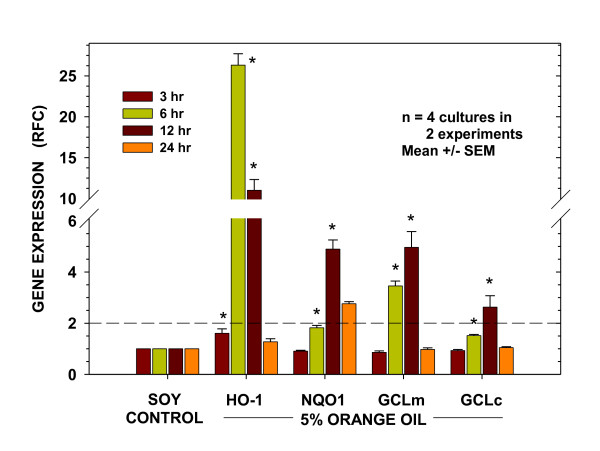
**Kinetics of orange oil-induced antioxidant gene expression**. Expression patterns of antioxidant genes *HO-1*, *NQO1*, *GCLm*, and *GCLc *in BEAS-2B cells at designated times following the 15 min treatment period. Data are presented as fold change from time-matched soy oil-treated controls after normalization to the expression of actin. Presented are results from two separate experiments. The dashed line indicates the 2-fold level of increased expression. Results are expressed as mean ± SEM. * indicates significantly different from time-matched soy oil-treated controls (P < 0.05) by Student's t test.

In order to confirm that the representative Nrf2-regulated antioxidant genes observed to be up-regulated following treatment with the mixed oil preparation were similarly activated by the orange oil alone, a set of experiments identical to those shown in Figure 3 were carried out using 5% orange oil in soy. The relative fold increase in expression for each of the genes at 3, 6, 12 and 24 hr post-treatment were compared to their corresponding time-matched soy oil controls. As seen in Figure 7, the kinetics and relative magnitudes of gene expression for each of the four genes in response to orange oil treatment were similar to those seen in response to the mixed oil preparation.

### Kinetics of orange oil-induced antioxidant protein expression

**Figure 8:**
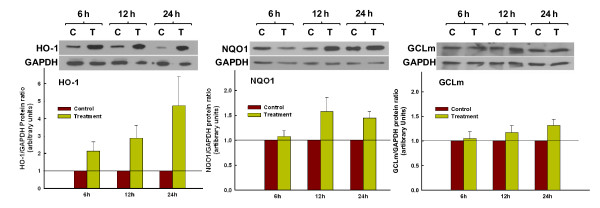
**Antioxidant enzyme synthesis in response to orange oil treatment**. Immunoblot analysis demonstrating expression of HO-1, NQO1 and GCLm proteins at 6, 12 and 24 hrs following 15 min treatment of BEAS-2B cells with the oil preparation or time-matched soy oil control. Representative blots from one of three separate experiments are shown above. Densitometric evaluations of each target protein blot normalized to its corresponding GAPDH for all three experiments are provided below. Bars represent mean ± SEM.

As confirmation that increased gene expression translated to increased protein synthesis, the time-course of increases in HO-1, NQO1 and GCLm was assessed at 6, 12 and 24 hr post-exposure to 5% orange oil by Western blot analysis in three separate experiments. As shown Figure 8, which provides representative blots from one of these experiments and summary data from all three, treated cultures were compared to time-matched soy treated controls at each time point. Treatment and control blot densities were normalized to GAPDH and are depicted below each pair of samples. Generally consistent with its early gene expression in response to 5% orange oil shown in Figure 7 and with the anticipated delay, HO-1 showed a rapid 2-fold increase in protein that was apparent by 6 hr and reached a greater than 4-fold level of increase by 24 hr. In contrast, NQO1 protein expression was less rapid, exhibiting a 1.5-fold increase that was not seen until 12 hr. GCLm showed a less pronounced increase in enzyme protein of 1.3-fold at 24 hr that followed its peak gene expression at 12 hr (Figure 7). These data, along with those of orange oil-induced nuclear translocation of Nrf2 and gene activation, offer strong evidence for orange oil as an effective activator of Nrf2 in respiratory epithelial cells.

### Inhibition of endotoxin-induced pro-inflammatory gene expression by orange oil

**Figure 9:**
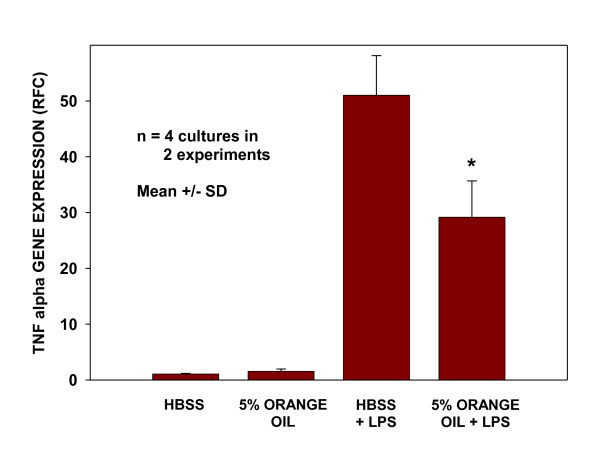
**Pretreatment with orange oil preparation attenuates LPS-induced expression of *TNFα***. Cells pretreated with HBSS or the orange oil in soy were challenged 12 hr later with LPS (3 ug/ml medium) or were left unchallenged. At 4 hr after LPS challenge, *TNFα *transcript levels were measured using real time RT-PCR. Data are presented as fold change from time-matched HBSS-treated controls after normalization to expression of actin. * indicates significantly different from cells HBSS pretreated and LPS challenged (P < 0.004) by Student's t test.

Following the same experimental procedure that had shown mitigation of the LPS-induced gene expression of the pro-inflammatory mediator, TNFα, by the mixed oil preparation, two separate experiments were undertaken to determine the extent to which this effect could be attributed to the orange oil component. BEAS-2B cell cultures were pretreated with 5% orange oil or with HBSS as control for 15 min and incubated for 12 hr prior to challenge with 3 ug/ml LPS. Cells were extracted for PCR analysis after 4 hours of LPS challenge. As shown in Figure 9, LPS challenge increased the expression of TNFα by approximately 50-fold in HBSS pre-treated cultures and 30-fold in those pre-treated with the orange oil. The significant 43% reduction in pro-inflammatory signaling produced by a single 15 minute treatment with orange oil 12 hours prior to LPS challenge indicates the presence of a significant and persistent modulatory effect of the oil on this inflammatory process. It also provides additional evidence that the presence of orange oil in the mixed oil preparation contributed to its observed anti-inflammatory activity in the nose.

### Nasal epithelial antioxidant gene expression in vivo

A study was carried out to determine if the up-regulation of antioxidant protective mechanisms observed in human respiratory epithelial cells in culture in response to oil treatment could be demonstrated in the nasal mucosa. We asked if the previously employed nasal aerosol spray administration of the oil mixture could demonstrably increase expression of the rapidly-responding *HO-1 *gene in the nasal mucosa 8 hr later. After obtaining informed consent, twelve healthy volunteers were recruited to participate in a protocol that took paired mucosal biopsies from both nares to allow subjects to act as their own controls, Biopsies were taken from the inferior medial turbinates of the right and left nasal passages prior to and 8 hours following oil treatment in the absence of ozone exposure. In 9 of the 12 subjects, *HO-1 *expression in mixed oil-treated turbinates increased by at least 1.5-fold above sham control and, in 6, expression was increased more than 2-fold at the 8 hour sampling time point (Table 3). That is, in all but 3 of 12 subjects, expression was detectably higher in oil-treated turbinates. Overall, paired analysis of the change in fold expression between sham-treated and oil-treated turbinates using the Wilcoxon Signed Rank Test demonstrated a significant difference (P < 0.03). There was a 2-fold median increase in gene expression in oil-treated nares compared to sham-treated nares across all subjects (Table 3). PCR analysis did not detect increases in any of the other target genes at this early time-point post-exposure. These data indicate that application of the original oil preparation containing orange oil has the ability to measurably increase antioxidant *HO-1 *gene expression in the human nasal mucosa and provides a potential mechanistic link between the antioxidant-associated *in vitro *data and the observation of decreased ozone-induced nasal inflammation.

**Table 3:** Nasal Epithelial HO-1 Gene Expression in Healthy Subjects

				Fold Change from Baseline*		
					
Subject#	Age (Yrs)	Gender		Sham Spray	Mixed Oil Spray	Mixed Oil Fold Change From Sham**
1	33	M		0.247	2.732	11.08
2	62	M		0.297	1.283	4.32
3	31	M		0.758	0.620	0.82
4	34	F		2.099	15.348	7.31
5	41	M		0.330	4.532	13.73
6	24	F		1.040	0.285	0.27
7	60	M		0.432	0.914	2.11
8	27	M		0.859	0.245	0.29
9	31	M		1.778	3.387	1.90
10	42	M		0.651	1.079	1.66
11	41	F		1.181	3.249	2.75
12	33	M		0.829	1.580	1.91
				
			Median	0.793	1.432†	2.01

*Data calculated as fold change after normalization to corresponding β-actin expression.

**Values less than 1.0 indicate a decrease.

†Significantly different from sham treatment (P = 0.027), Wilcoxon Signed Rank Test.

## Discussion

The present study was designed to determine if naturally-occurring oils with antioxidant properties could be utilized to provide protection against proinflammatory challenges to the upper respiratory tract. The mechanisms involved with such protection would likely include their direct ability to scavenge ROS that arise as a consequence of toxicant exposure in the nasal epithelial mucosa. In addition, the presence of molecular constituents within these oils could presumably have an effect on antioxidant gene expression within mucosal cells, contributing further to their innate defense against agents that induce inflammation through oxidant-related pathways.

Exposure to ozone has long been known to lead to an inflammatory response in the upper and lower respiratory tracts characterized by the influx of PMNs [4,41]. Under conditions of controlled exposure of subjects to 0.25 ppm ozone for 2 hours in the present study, this response was observed in the upper respiratory tract. Pretreatment of the nasal passages by aerosol spray with a natural oil preparation inhibited the inflammatory response. Because of the reactive target that the antioxidant oil mixture and the soy oil carrier (diluent) might present to inhaled ozone or its reactive products, some degree of protection could have been provided by a simple "barrier" effect [45]. This would likely be greatest at times early in the exposure period prior to a reduction in surface oil levels that might result from nasal mucociliary clearance [46]. If this were the sole mechanism involved, it might be expected that administration of the oil would reduce or, at best, eliminate the ozone-induced influx of inflammatory cells. However, the data demonstrated that PMN levels in the nasal lumen at the 18 hour post-exposure time point were significantly reduced below those observed at baseline in the pre-exposure samples. This observation suggests that proinflammatory signaling was abrogated in the nasal mucosa by the treatment, possibly through mechanisms involving increased intracellular antioxidant activity leading to reduced inflammatory drive. In addition, it indicates that this activity persisted in the tissue, at least to the sampling point 18 hours after the ozone exposure period.

Ozone has been shown to stimulate the influx of PMNs into the airway lumen as early as 1 hr following exposure [47] suggesting very rapid initiation of pro-inflammatory signaling. In the present study, the effectiveness of the oil in reducing inflammation following exposure to ozone could have had several temporal components. In addition to the likely ROS scavenger effect of the mixed oil preparation previously described, pre-treatment of the nasal mucosa prior to exposure could have stimulated early activation of intracellular antioxidant pathways to increase baseline cellular protection. Involvement of antioxidant genes with varying kinetic profiles of activity could provide both immediate and more prolonged antioxidant capacity. The activity of such a mechanism would be consistent with the observed early induction of the rapidly-responding enzyme, HO-1. *HO-1 *gene expression was increased by 3-fold in cultured cells within 3 hours of oil treatment and was also found to be elevated in the nasal epithelium of naïve subjects 8 hr following administration of the mixed oil preparation. Furthermore, the demonstration of related antioxidant genes with delayed expression kinetics in the cell culture studies provides the mechanistic basis for a sustained antioxidant effect.

The four antioxidant genes investigated in this study, *HO-1*, *NQO1, GCLc*, and *GCLm*, are known to have a common antioxidant response element (ARE) in their promoters and are expressed in an Nrf2-dependent manner. The basic leucine zipper (bZip) transcription factor, Nrf2, acting via an antioxidant/electrophile response element, regulates the global expression of a family of antioxidant enzymes and functions to maintain cellular redox homeostasis [48]. The present investigation of the mixed oil preparation demonstrated that the Nrf2-associated gene and protein expression observed was predominantly associated with the orange oil component. Nrf2 activation by that oil was confirmed in a battery of cell culture studies that demonstrated activation kinetics, dose-dependency, translocation of Nrf2 protein to the nucleus, and the gene and protein expression of Nrf2-activated antioxidants. Although the focus of the present study was directed toward the Nrf2 system, the possibility remains that other components of the mixed oil preparation activated additional antioxidant or anti-inflammatory-associated transcription factors that contributed to the observed reduction of nasal mucosal inflammation.

Among the antioxidant genes studied, *HO-1 *induction in response to oil treatment was the most rapid and dramatic. Heme oxygenase catalyzes the rate limiting steps of heme oxidation to biliverdin, carbon monoxide and iron. Biliverdin is rapidly converted to bilirubin, a potent endogenous antioxidant. Three isoforms of heme oxygenase have been reported: the inducible HO-1 and the constitutively expressed HO-2 and HO-3. An increasing number of studies implicate HO-1 in the regulation of inflammation. The induction of HO-1 has been demonstrated in many models of lung injury including hyperoxia, endotoxemia, bleomycin, asthma, acute complement-dependent lung inflammation, and heavy metals [49,50].

In an *in vitro *model of oxidative stress using pulmonary epithelial cells stably transfected to over-express HO-1, Lee *et al*. [51] demonstrated that these cells exhibited increased resistance to hyperoxic cell injury. In studies by Petrache *et al*. [52] and Soares *et al*. [53], HO-1 also prevented TNF-α-mediated apoptosis in fibroblasts and endothelial cells, respectively. Such findings further underscore the importance of HO-1 in cytoprotection and the potential prophylactic benefits of its up-regulation.

Otterbein and colleagues [54-56] have demonstrated that HO-1 induction correlated with cytoprotection against oxidative stress *in vivo*. Using hyperoxia as a model of acute respiratory distress syndrome in rats, they demonstrated that the exogenous administration of HO-1 by gene transfer could confer protection against oxidant-induced tissue injury. Adenoviral gene transfer of HO-1 (Ad5-HO-1) into the lungs of rats resulted in increased expression of HO-1 and, importantly, induced a marked resistance to hyperoxic lung injury [56,57]. Rats treated with Ad5-HO-1 showed reduced levels of hyperoxia-induced pleural effusion, neutrophil alveolitis, and bronchoalveolar lavage protein leakage. Furthermore, rats over-expressing HO-1 showed increased survivability in the presence of hyperoxic stress versus those treated with the vector control virus [56,57].

Another of the antioxidants observed to undergo up-regulation was NQO1, an enzyme primarily expressed in tissues requiring a high level of antioxidant protection, such as the epithelial cells of the lung, breast, colon, and vascular endothelium. Expression of *NQO1*, suggests that this molecule may play a key role in establishing the antioxidant capacity in these cells [58]. Oxidant pollutants, including diesel exhaust particles, induce NQO1 expression which plays a role in mitigating pollutant-enhanced IgE responses [58,59]. Furthermore, over-expression of phase II enzymes, including NQO1, inhibited IgE production and supports the concept that chemical up-regulation of these enzymes may represent a chemopreventative strategy in airway allergic diseases [58,59]. Thus, the oil-induced inductions of NQO1 and related antioxidants may have broader implications for protection of the respiratory mucosa, extending to pollutant-related pro-allergenic effects in susceptible individuals.

Cellular antioxidant defenses can counter inflammation by limiting the levels of ROS generated. Expression of genes involved in glutathione biosynthesis (i.e. *GCLc*, and *GCLm*) was significantly up-regulated in response to oil pretreatment. Further, pretreatment of BEAS-2B cells with the mixed oil preparation or orange oil alone was observed to greatly suppressed *TNFα *activation in response to LPS treatment. Lower levels of glutathione have been reported to augment activation of the proinflammatory transcription factor, NF-κB [60], consistent with our previous report of a protective role of glutathione peroxidase in LPS-induced septic inflammation [61]. In addition, induction of HO-1 may exert anti-inflammatory functions through the generation of carbon monoxide and has been shown to inhibit the LPS-induced expression of proinflammatory cytokines [50].

The results of the present study indicate that the mixture of natural oils was capable of reversing the nasal inflammatory response to ozone exposure in healthy human subjects in a manner that persisted for up to 18 hours. In human airway epithelial cells in culture, short duration (15 min) treatment with the mixture resulted in activation of Nrf2 and increases in the expression of several representative antioxidant genes with both rapid response and late activation profiles. The effectiveness of the short treatment duration was surprising, given the significantly longer period identified in our preliminary studies (12 hr) and utilized by others [62] (24 hr) to elicit robust activation of Nrf2-dependent enzyme systems using the water-soluble Nrf2 activator, sulforaphane. It may be that the oil-based preparation allows for rapid integration of the active component(s) into the cell membrane which then acts as a repository for sustained release into the cell. It will be of interest to determine if the presence of a lipid-based carrier plays a role in increasing the duration of antioxidant pathway activation. Consistent with the observed up-regulation of Nrf2-activated antioxidant genes in cultured airway epithelial cells, treatment of test subjects with the oil mixture by nasal spray induced detectable increases in nasal mucosal HO-1 gene expression. Although the easiest of the genes to assess *in vivo *due to its rapid- and high-responding profile, the increased expression of this gene *in vivo *provides support for the notion that the mechanism of action identified for the oil in the cell culture studies is associated with the protection observed in the nasal ozone intervention study.

Although seemingly unrelated in terms of their sources and the nature of their interactions with the respiratory system, many environmental challenges share the development of cellular oxidative stress as a common pathway for cell activation, inflammation and, in some cases, cytotoxicity. Such challenges include virus and bacterial infection [10,63], allergen challenge [64], and exposure to common gaseous and particulate air pollutants [65,66], tobacco smoke [67], and bacterial endotoxin [68]. It is important to note that many of these diverse exposures occur in combination and may synergize to produce greatly amplified responses within epithelial cells of the respiratory tract. For example, in a study of human bronchial epithelial cells in culture, it was observed that the consequences of oxidant stress induced by the oxidant pollutants ozone or nitrogen dioxide in combination with rhinovirus infection resulted in release of the proinflammatory mediator, IL-8, at levels as much as 2.5-fold greater than those predicted by the individual exposures [3]. Thus, targeting oxidant-driven pathways leading to inflammatory responses in the upper respiratory tract may offer a means to provide cytoprotection against a range of environmental challenges to those tissues. Such antioxidant strategies may be especially beneficial in individuals who have reduced or absent phase II enzyme activity, as may result from certain genetic polymorphisms. Furthermore, the unique mechanisms of action afforded by natural oil-derived preparations may offer opportunities to broaden therapeutic approaches for those individuals who are poorly responsive to current treatments.

## Conclusions

The present study demonstrates that aerosol spray delivery of a mixture of natural oils described to have antioxidant properties was able to abrogate the inflammatory response to the oxidant pollutant, ozone, in the nasal passages of healthy subjects. This oil preparation stimulated the expression of several Nrf2-regulated early and late responding antioxidant genes in human respiratory epithelial cells in culture. The most rapidly-responding of these genes, HO-1, was determined to undergo increased expression in the nasal tissues of subjects treated with the oil mixture. Nrf2 activation was confirmed in cell culture experiments and was associated with the orange oil component. In total, these novel data offer evidence that selected oil-based agents may utilize mechanisms beyond direct ROS scavenging, such as the activation of intracellular antioxidant pathways, to strengthen anti-inflammatory protection within the nasal epithelial mucosa. The ability of oil pre-treatment to inhibit the pro-inflammatory action of subsequent bacterial endotoxin exposure of human respiratory epithelial cells suggests the potential usefulness of such a preparation in mitigating a broader array of inflammatory and cytotoxic exposures to the upper respiratory system.

The upper respiratory epithelial mucosa, at the juncture of the external and internal environments, plays a key role in the success of adaptive responses to oxidant-stress related challenges, such as those associated with pollutant exposures, microbial infections and allergic challenge. The identification of naturally-occurring, well-tolerated and potent activators of cytoprotective mechanisms within these cells, such as the oil preparation investigated here, will expand our ability to develop new tools for preventive and therapeutic intervention at this critical respiratory interface.

## Abbreviations

AD5: adenovirus 5; ARE: antioxidant response element; COPD: chronic obstructive pulmonary disease; GCLc: glutamate cysteine ligase - catalytic subunit; GCLm: glutamate cysteine ligase - modulatory subunit; HBSS: Hank's balanced salt solution; HO-1: heme oxygenase-1; IgE: immunoglobulin E; LPS: lipopolysaccharide bacterial endotoxin; NQO1: nicotinamide adenine dinucleotide phosphate:quinone oxidoreductase 1; Nrf2: nuclear factor erythroid-2 related factor 2; PMNs: polymorphonuclear leukocytes; RFC: relative fold change; ROS: reactive oxidant species; TNF*α*: tumor necrosis factor alpha.

## Competing interests

The authors declare that no competing interests exist for any of the authors of this work and that partial funding by the company that supplied the test oils had no influence on the outcome or publication of the work.

## Authors' contributions

MG was responsible for designing and carrying out the majority of in vitro studies and molecular biological assessments of cell and tissue samples, assisted in data analysis, and the construction of figures. AS contributed to study design, conducted many *in vitro *studies, assisted in data analysis, and wrote key sections of the manuscript. KM supervised and conducted human subject studies and assisted in human data analysis. CR carried out nasal biopsies and assisted in human subject studies. VS carried out or assisted with assays and cell culture work. SB and ES conceived of and contributed to the design of the studies, supervised data analysis and contributed to writing and editing the final manuscript. All authors read and approved the final manuscript.
